# Tumour heterogeneity and evolutionary dynamics in colorectal cancer

**DOI:** 10.1038/s41389-021-00342-x

**Published:** 2021-07-16

**Authors:** Dedrick Kok Hong Chan, Simon James Alexander Buczacki

**Affiliations:** Nuffield Department of Surgical Sciences, Medical Sciences Division, University of Oxford, Oxford, UK

**Keywords:** Tumour heterogeneity, Colorectal cancer

## Abstract

Colorectal cancer (CRC) has a global burden of disease. Our current understanding of CRC has progressed from initial discoveries which focused on the stepwise accumulation of key driver mutations, as encapsulated in the Vogelstein model, to one in which marked heterogeneity leads to a complex interplay between clonal populations. Current evidence suggests that an initial explosion, or “Big Bang”, of genetic diversity is followed by a period of neutral dynamics. A thorough understanding of this interplay between clonal populations during neutral evolution gives insights into the roles in which driver genes may participate in the progress from normal colonic epithelium to adenoma and carcinoma. Recent advances have focused not only on genetics, transcriptomics, and proteomics but have also investigated the ecological and evolutionary processes which transform normal cells into cancer. This review first describes the role which driver mutations play in the Vogelstein model and subsequently demonstrates the evidence which supports a more complex model. This article also aims to underscore the significance of tumour heterogeneity and diverse clonal populations in cancer progression.

## Epidemiology of colorectal cancer (CRC)—a global disease

Annually, 1.8 million new cases of CRC are diagnosed worldwide, with close to 900,000 dying due to late-stage presentation or advanced disease [1]. As a result, CRC is the third most common cancer diagnosed, and the second leading cause of cancer mortality [2]. CRC is considered to be a disease that afflicts developed countries, with the highest incidence of cases occurring in parts of Europe including Norway and the Netherlands, Australia, North America, as well as Asian countries including Japan, the Republic of Korea, and Singapore [2]. There has also been an increasing trend of younger patients with CRC in countries such as Denmark, Australia, New Zealand, and the UK, where patients aged less than 50 years make up 3% of the incidence of CRC [3]. Although the incidence of CRC increases in tandem with the developmental status of populations [4], a rising incidence but declining mortality rate is often observed in countries at a higher level of development, while higher case fatality rates may be observed in countries with a smaller burden of disease due to poorer access to healthcare infrastructure and insufficient resources for screening and detection [2]. The trajectory of CRC mortality is therefore expected to further worsen in lower- and middle-income countries found in parts of Latin America, the Caribbean, and Asia [5].

The association between a country’s developmental status and an increased incidence of CRC has been attributed to changes in lifestyle that come with increased affluence. This includes a diet high in red and processed meats [6], excessive alcohol consumption [7], obesity [8], diabetes mellitus [9], as well as a sedentary lifestyle [10]. Together with non-modifiable risk factors for CRC such as increased age and male gender, these environmental factors likely account for a large proportion of the increased risk at a population level but do not explain the pathogenesis of CRC at an individual level, for which a molecular basis must be sought. Environmental factors must interact with genetic factors, giving rise to CRC.

## Genetic mechanisms of CRC

CRC carcinogenesis proceeds via three main genetic pathways. Cancers can arise as a result of chromosomal instability (CIN), microsatellite instability (MSI), or through the serrated neoplasia pathway.

### CIN

CIN is the most common pathway from which CRC arises, occurring in 65–70% of cancers [11], and is characterised by widespread copy number alterations, indels, translocations, amplifications, and loss of heterozygosity (LOH). These genetic abnormalities arise as a result of dysfunctional chromosomal segregation, disordered telomere viability, and ineffective DNA-damage response machinery [12].

*FBXW7* is a commonly mutated CRC gene found in ~7.5% of cases [13]. Centromere protein A (CENP-A) is essential for normal centromere and kinetochore function, failing of which leads to chromosomal missegregation [14]. Loss of *FBXW7* results in CENP-A phosphorylation mediated by cyclin E1 and CDK2, leading to a reduction of CENP-A levels at centromeres. In CRC, *FBXW7* mutations lead to lagging chromosomes and chromosomal bridges which contribute to CIN [15].

Other modalities of CIN in CRC include a high frequency of LOH at chromosomes 1, 5, 8, 17, and 18 [16], as well as defects in genes such as *RAD51*, which are responsible for homologous recombination in DNA double-stranded damage [17].

### MSI

MSI is responsible for ~15% of CRC [18]. The mismatch repair (MMR) system functions by correcting erroneous bases which occur during DNA replication and recombination and plays a critical role in ensuring these mutations do not get propagated to daughter cells [19].

More recently, the interplay between MSI CRC and endogenous immunogenic responses has been described. Regions of high genetic instability, of which MSI CRC is characteristic, induce a cytotoxic T cell response due to the appearance of frameshift mutation-generating neoepitopes [20]. In patients with CRC, the production of immune checkpoints, such as PD-L1 and CTLA-4 incite an anti-tumour immune response by allowing tumour cells to escape T-cell detection [21]. Immune checkpoint inhibitors such as anti-CTLA-4 and anti-PD-1 antibodies have been developed. These antibodies block T-cell checkpoints and allow for an endogenous immune response against tumour cells. Results from the KEYNOTE-177 trial demonstrated that the anti-PD-1 monoclonal antibody pembrolizumab outperformed standard first-line therapy in MSI metastatic CRC with an improvement in progression-free survival from median 8.2 months to 16.5 months (95% confidence interval 0.45–0.80; *p* = 0.0002) with fewer adverse effects [22].

### Serrated neoplasia

The serrated neoplasia pathway is responsible for 15–25% of CRC [23] and gives rise to serrated adenomas. It is named for its characteristic histologic appearance of stellate crypt folding patterns which appear as serrations on hematoxylin and eosin staining [24]. This pathway is characterised by a unique sequence of molecular and genetic changes, which may be tracked by histologic features. An activating V600E mutation in BRAF, which regulates the mitogen-activated protein kinase pathway, is the putative initial mutation event [25]. *BRAF* mutations result in constitutive activation of the mitogen-activated protein kinase-ERK pathway which leads to uncontrolled cellular proliferation and division [26].

*BRAF* activation results in widespread methylation of CpG islands, concentrated clusters of the cytosine residue followed by a guanine nucleotide, referred to as the CpG island methylator phenotype (CIMP) [27]. These CpG islands predominantly appear in the promoter region of genes [28], and upstream of important tumour suppressor genes. Mutant BRAF is known to upregulate the transcriptional repressor MAFG [29], which in turn recruits BACH1, CHD8, and DNMT3B to result in a hypermethylation phenotype [30]. CRCs developing via the serrated neoplasia pathway are more aggressive, with progression from adenoma to cancer within 1–3 years [31].

## Intra-tumoural heterogeneity

Vogelstein proposed a linear accumulation of driver mutations, each conferring an additional survival advantage relative to surrounding cells. Genes that harbour driver mutations include *TP53*, *APC*, *SMAD4*, and *KRAS*. This linear model would be expected to give rise to largely homogeneous tumours, with each cancer cell in the tumour possessing the same mutations. Studies that have sampled multiple regions of the same cancer have yielded results that suggest otherwise. In a study by Losi et al. [32], among ten CRC cases, each with 9–14 areas of sampling, 90% of patients demonstrated intratumoural heterogeneity (ITH) on single-strand conformation polymorphism (SSCP) analyses. Moreover, in two patients, different polymorphisms of the *KRAS* and *TP53* mutations were detected within the same tumour. More recent experiments have used multiregional sequencing analysis (MRA) to demonstrate marked heterogeneity within tumours. One approach adopted by Saito et al. to perform MRA was to use whole-exome sequencing (WES) at disparate regions of the tumour, allowing for somatic mutations to be classified as either “ubiquitous” or “heterogeneous”, depending on whether that particular mutation was present in all regions of the samples, or only in a proportion of sampled regions respectively [33]. These studies have demonstrated that on average, each tumour possesses about 75 different mutations, with approximately 15 of such mutations classified as being driver mutations [34]. In a study by Uchi et al. [35], samples from 9 CRC patients underwent WES. Their results demonstrated 5068 ubiquitous mutations, but also 3107 mutations that were subclonal. In addition, 1362 of the subclonal mutations were unique to single samples. Taken together, these results suggest that CRC does not progress via a linear accumulation of driver mutations and subsequent clonal sweeps. Instead, an alternative process that results in the variegation of mutations is more likely. Subsequent paragraphs will expand on the clinical implications of heterogeneity and review contemporary mechanisms of heterogeneity.

### ITH poses challenges in the clinical management of CRC

Recently, an association between the level of ITH and prognosis has been demonstrated. By using Shannon’s Index to evaluate the variant allele frequencies (VAFs) of 381 cancer-related genes, Oh et al. were able to generate a tumour heterogeneity index (TH index) [36]. High-TH index cases correlated with cancers at a more advanced stage. Survival analyses of TH indices also demonstrated a significant association between high-TH and low-TH patients with regards to progression-free survival. Transcriptomic heterogeneity may also preclude accurate prognostication and management of CRC on diagnosis and has been used as a marker of poor outcomes. Commercial assays are available which attempt to utilise subsets of gene-expression assays to prognosticate CRC, such as the Oncotype DX 12-gene RT-PCR assay (Genomic Health, USA) [37], and the ColoPrint 18-gene microarray-based classifier (Agendia Inc., USA) [38]. However, the complexity of transcriptomic heterogeneity is evident when such arrays have been shown to provide discordant assessments in 48% of cases on the risk of disease progression when compared with standard clinical criteria.

A classification system was developed by Sadanandam et al. based on the gene expression profiles of CRC tumours. In this classification, one subtype is the transit-amplifying (TA) subtype, a heterogeneous group characterised by variable expression of Wnt-target and stem cell genes [39]. Heterogeneity within the TA group has been found to be associated with different responses to the anti-epidermal growth factor receptor (EGFR) drug cetuximab. CRC which highly expressed TA signature genes had a longer progression-free survival compared to CRC with a reduced expression [40].

ITH is also a possible mechanism for recurrence and metastasis. Cetuximab is effective in killing cells with wild-type *KRAS*, but not mutant *KRAS*. Using DiFi and Lim1215 CRC cell lines which both overexpress EGFR but are wild-type for *KRAS*, Misale et al. demonstrated that continuous cetuximab administration resulted in resistance of the cell lines to cetuximab over time [41]. Sequencing analysis of the cetuximab-resistant cell lines showed that they had acquired KRAS G12D or KRAS G12R mutations. The authors were able to validate two processes by which resistance was developed. By deep sequencing of the parental cell lines, they uncovered that 0.2% of the parental Lim1215 CRC cells harboured the mutant KRAS G12D genotype which eventually became predominant, leading the authors to conclude that cetuximab exerted a selection advantage to the small proportion of cells that were resistant, and was able to expand and repopulate the tumour when sensitive cells were killed. The authors also validated another possibility that the mutations arose de novo. By cloning a homogeneous population of *KRAS* wild-type Lim1215 cells confirmed on mass spectrometry-based genotyping and 454 pyrosequencing analyses, cells passaged in increasing concentrations of cetuximab eventually became resistant to cetuximab and exhibited upregulation of KRAS. Importantly, in patients who had tumour biopsies performed before and after the initiation of cetuximab, this observation of initially wild-type *KRAS* tumours acquiring a resistant phenotype and becoming mutant *KRAS* was reproduced [41]. These findings suggest that testing for *RAS* mutation status may need to be performed during the treatment course with cetuximab. While patients may gain resistance to cetuximab through the clonal selection of *RAS* mutant subclones during cetuximab therapy, the reverse has similarly been observed following even short breaks from cetuximab therapy, rendering previously resistant tumours sensitive again [42].

### ITH is dynamic through the adenoma-carcinoma sequence

**Fig. 1:**
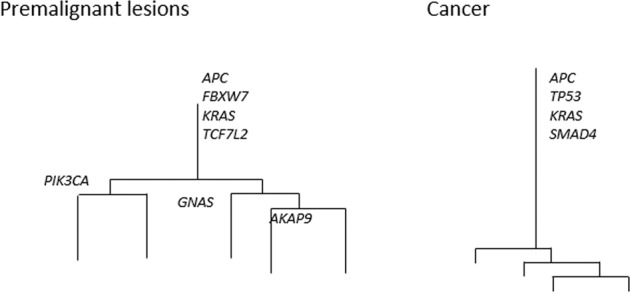
Representative dendrogram showing differences in the distribution of mutations in premalignant lesions, and cancer. In (**a**) premalignant lesions, the trunk tends to be shorter with multiple branches, suggesting the presence of multiple subclones. In (**b**) cancer, the trunks are longer, demonstrating that cancer cells tend to be clonal. These dendrograms are adapted from Cross et al. [43].

Interestingly, ITH does not remain static through its progression from adenoma to carcinoma. In order to demonstrate the degree of ITH within a tumour, evolutionary trees have been used to represent the frequency of ubiquitous and heterogeneous mutations within tumours (Fig. 1). Ubiquitous mutations which are present throughout the tumour correspond to trunks on the evolutionary tree, while heterogeneous mutations which only occur in a subclone of the tumour are represented by branches. In a comparison of premalignant lesions and advanced CRC lesions, Saito et al. noted that premalignant lesions tended to have shorter trunks and more branches, while advanced CRC lesions had longer trunks and shorter branches [33]. These findings demonstrate that a greater proportion of mutations within CRC lesions were ubiquitous in nature, whereas more mutations in premalignant lesions tended to be heterogeneous. In pre-cancerous colonic lesions, 25 out of 51 driver mutations were branch mutations, whereas in advanced CRC only 10 out of 45 driver mutations were branch mutations. VAF within branches were also notably higher in premalignant lesions than in CRC. Similar findings were recapitulated in a study by Cross et al. [43], which also compared premalignant lesions with CRC. The authors noted that there were no differences in the median number of somatic single-nucleotide alterations between premalignant lesions and CRC, but that premalignant lesions tended to have less ubiquitous mutations and a larger proportion of heterogeneous mutations compared with CRC. Notably, the presence of key driver mutations was present in both clones and subclones in premalignant lesions but tended to be exclusively clonal in CRC lesions. Subclones with a survival advantage in premalignant lesions, therefore, outcompete other subclones and become established as the dominant clone in its progression to cancer.

### Evolution and expansion of single nucleotide variations

The evolutionary mechanism which brings about ITH remains an area of key interest. The normal colonic epithelium is known to already harbour a range of mutational processes, including base-substitutions and indels. A study that performed whole-genome sequencing on 571 normal colonic crypts supplemented with targeted sequencing of 90 known CRC genes found the presence of driver mutations present in the normal epithelium [44]. Positive selection of the genes *AXIN2* and *STAG2,* as well as highly specific hotspot mutations in *PIK3CA*, *ERBB2*, *ERBB3*, and *FBXW7*, suggested that these genes harboured driver mutations found in normal colonic epithelium. Another study by Nicholson et al. demonstrated both *KRAS* and *STAG2* mutations could be identified in normal colonic epithelium. These mutations conferred a selective advantage to clones which allowed for expansion 155- and 13-fold in excess of neutral mutations in *KRAS* and *STAG2* mutations respectively [45].

Intriguingly, premalignant lesions have been found to harbour the same driver mutations as CRC, yet do not exhibit a malignant phenotype. An evaluation of driver mutations in small subcentimetre colorectal polyps demonstrated that out of 48 polyps, 32 (67%) had *APC* mutations, 7 (15%) had *KRAS* mutations, and 4 (8%) had *TP53* mutations [46]. Almost a third (15/48) of all polyps had already harboured at least two CRC driver mutations. Another study that investigated 34 adenoma and carcinoma pairs using a 100-gene CRC-specific panel showed that 48% of pairs did not acquire any further new mutations in the transition from adenoma to carcinoma [47]. Together, these findings suggest that the molecular genotype does not necessarily translate into a malignant phenotype, hinting at the presence of alternative processes which might translate a particular genotype into a malignant phenotype and that a stepwise evolutionary process was insufficient to completely explain the cancer phenotype.

**Fig. 2:**
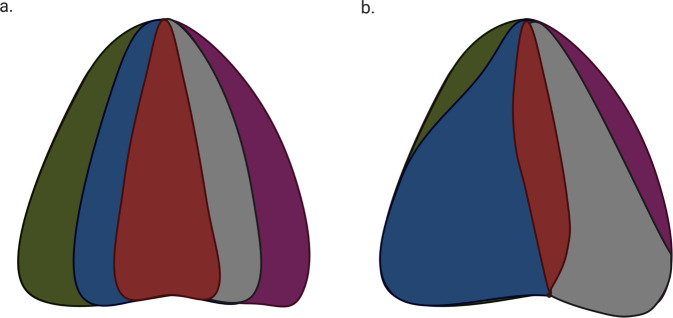
Two different evolutionary models are presented here, with each colour representing a subclonal population. In (**a**) the big bang model, multiple subclonal populations are seen early and followed by a period of neutral evolution. In (**b**) the Darwinian model, subclones with a selective advantage outgrow other subclones.

The observation of marked ITH and the presence of driver mutations even in premalignant lesions suggests that evolution by Darwinian mechanics is unlikely. Modelling by Ling et al. comparing Darwinian mechanics and non-Darwinian mechanics in hepatocellular carcinoma showed that the extremely high genetic diversity made Darwinian evolution unlikely given that Darwinian evolution favours a reduction in genetic diversity [48]. When nearly 300 regions of a tumour were sequenced, the authors found more than 100 million coding region mutations. This high volume of genetic diversity does not support Darwinian evolution as the magnitude of mutations would have been expected to be several magnitudes lower. Sottoriva et al. introduced the concept of a big bang model of CRC tumorigenesis, in which tumours grow exponentially at a primordial phase as early as at the precancerous stage, generating the marked ITH and diverse subclone populations present in CRC [49] (Fig. 2). By introducing a spatial dimension in the evaluation of both copy number alterations as well as genomic sequencing, the authors were able to demonstrate that some subclonal, or private, mutations were variegated at spatially distinct locations of the tumour, yet possessed a trajectory indicating that they arose from a single origin. In addition, by analysing copy number heterogeneity of physically adjacent cells within tumour glands, the authors noted marked diversity and a high degree of variability of copy number which suggested that selective sweeps, more likely in Darwinian evolution, did not occur.

Following the big bang, tumours are likely to undergo a period of evolutionary stasis, referred to as neutral evolution. During this period, the subclones attain an optimal phenotype, such that any further mutation results in negative selection [50]. Given that not all premalignant lesions progress to cancer, and that such premalignant lesions greatly outnumber cancers, this suggests that there is often a prolonged period of dormancy [51]. Martincorena et al. approached the evolutionary dynamic in cancer by calculating the ratio of non-synonymous mutations (N) to synonymous mutations (S) amongst cancer genes. Given that synonymous mutations reflect an evolutionary neutral mutation background, a dN/dS ratio above 1 would reflect positive selection, implying a driver mutation, while a dN/dS ratio below 1 would suggest negative selection [52]. By analysing a large dataset of multiple genes across different cancer types, the authors found that most genes were not under negative selection, and yielded dN/dS values which were just above one, suggesting that neutral evolution was likely the most prevalent evolutionary dynamic in cancer.

In silico modelling has been used to substantiate the presence of neutral drift. Williams et al. postulated that should tumour growth be defined entirely by neutral evolution, the number of mutations within a fraction of the tumour should be proportional to an inverse of that fraction of cells [53]. Using data from the cancer genome atlas (TCGA) and a variety of cancer types, the authors concluded that about a third of cancers conformed to their hypothesis and were neutrally evolving. These in silica findings have however been subjected to some debate. Bozic et al. argue that detecting drivers deviating from neutral evolution is challenging as most drivers are unlikely to be present within a detectable range [54]. When the tumour size is small, the presence of driver mutations with a selection advantage is present in such small amounts that its frequency is likely to approximate 0. Conversely, when the tumour has expanded, the driver mutations are likely to be clonal and dominate the tumour with a frequency approaching 100%. As such, there is the risk of a skewing of mutations towards being neutral with a frequency of 0, or clonal, with a frequency of 1. It is likely that these findings will only be resolved through a directly observed experiment into the evolutionary trajectory of mutations within a tumour.

### Copy number variations (CNVs) can also give rise to ITH

While the above section focused on the generation of single nucleotide variations and evolutionary mechanisms, CNVs are an equally important characteristic of CRC, and may also contribute to ITH. In a study in which 27 patient CRC samples underwent DNA sequencing, ITH was contributed predominantly by the burden of CNVs present, while concordance in the loci of mutations in driver genes such as *APC*, *KRAS*, *NRAS*, *PIK3CA* and *SMAD4* was instead observed [55]. In another study by Sakimura et al., CNVs were found to be more frequent in advanced-stage cancer compared with early CRC amongst 409 cases in TCGA [56]. Interestingly, the authors also demonstrated an inverse relationship between arm-level CNVs and the number of single nucleotide variations in CRC. This association between advanced cancer and CNVs was also observed in a cohort of patients who had colorectal liver metastases [57]. Genome-wide CNV burden, as well as intermetastatic CNV heterogeneity, defined as a measure of CNV between metastatic lesions found in the same patient, was associated with poorer patient prognosis. In patients with *TP53*-mutated or *RAS/BRAF*^*V600E*^-mutated tumours, the addition of a heavy CNV burden was independently associated with worse outcomes, providing evidence that single nucleotide variations and CNVs are likely to interact in CRC.

While mechanisms resulting in CNVs have been discussed earlier in this paper, one mechanism relevant to CRC is punctuated copy number evolution (PCNE). By tracking the change in VAFs of single nucleotide variants, Cross et al. were able to approximate the time of copy number alteration. This was estimated on the basis that SNVs acquired prior to a copy number gain would increase in VAF whereas SNVs acquired following a copy number change would be present on only one allele and therefore have a lower VAF. The authors were able to demonstrate clustering of copy number alteration timings for a number of SNVs, suggesting that a punctuated evolution existed [43]. Intriguingly, studies performed on breast cancer have shed light on evolutionary activity occurring following the PCNE event. Single-cell DNA sequencing (scDNA-seq) of 9765 breast cancer cells confirmed the presence of multiple subclones with similar copy number profiles [58]. Mathematical modelling suggested that the evolution of copy number persists following the PCNE event and that a transient period of increased CIN occurs immediately after the PCNE event before stabilising to a gradual evolution model which persists during the growth of the tumour.

Mechanisms of CNV are likely to complement somatic mutations which interact to drive positive selection in tumour subclones. This interaction between subclonal CNVs and somatic mutations was investigated by Watkins et al [59]. Interestingly, the propensity for arm-level copy number alterations was significantly correlated with the imbalance of tumour-suppressor genes and oncogenes encoded on each chromosomal arm. A computational model to determine the timing of somatic copy-number alteration revealed patterns of CNV. While some copy number alterations such as loss on chromosome 17p13.3–q11.2, which encompasses *TP53*, were early alterations amongst most cancers, some alterations were unique to specific cancers. In CRC, the earliest copy number alterations involve chromosomes 9p24.3-p21.1 and 6p21.1. The overall evolution of tumours is likely to be shaped by both CNVs and somatic mutations.

### Unique features of ITH in microsatellite unstable CRC

Genomic instability characterised by DNA MMR deficiency affects coding mononucleotide repeats in tumour-suppressor genes. Indels in tumour-suppressor genes result in frameshift mutations which generate unique frameshift peptides and are a source of neoantigens [60,61,]. In MSI cancers, these neoantigens are readily recognised by the host immune system, and result in highly immunogenic tumours, accounting for the unique feature of immunoediting [62]. By analysing a large number of genes susceptible to a mutation in MSI CRC, commonly occurring frameshift peptides were identified, and immunogenic profiles for such peptides could be modelled. In this paper, the authors established that HLA class I-mediated antigen processing of neoantigens occur, and MSI clones with immunogenic frameshift peptides may undergo immunoediting by T-cells. Conversely, immune evading mechanisms such as alteration of the HLA class I heavy chain may affect immunoediting by T-cells, and in turn, affect the evolutionary trajectory of the tumour.

Another study attempted to define the tumour evolution of microsatellite unstable tumours by plotting phylogenetic trees of subclones on the basis of microsatellite loci [63]. The authors noted that microsatellite loci tended to shorten in MMR deficient cells over time, as a result of novel mutations introduced, resulting in subclone divergence. One application of this finding in the clinical setting is the use of patterns of microsatellites to determine response or relapse during the clinical course.

Investigations into tumour evolution and heterogeneity in serrated adenoma subtype CRC however remain forthcoming, and elucidating unique features in this subgroup of tumours could be grounds for further research.

### Transcriptional profiles are an additional source of heterogeneity

While most studies have focused on the genetic heterogeneity of CRC, another source of heterogeneity may arise from the transcriptional profile of cancer cells. One such gene-expression based subtyping is the CMS classification system [64]. Consensus molecular subtype (CMS) is further evidence that there is significant transcriptomic heterogeneity amongst CRC. In the CMS classification, four subtypes of CRC can be described based on their transcriptomic profile. CMS4, present in a quarter of CRC, demonstrates prominent TGFβ activation, stromal invasion and angiogenesis, which is associated with the worst prognosis. A comparison of CMS subtypes with prognosis demonstrated a worse overall survival in CRC classified as CMS 4 (hazard ratio 1.7, *p* = 0.021) [65]. Understanding CRC heterogeneity based on this CMS profile may be useful in guiding therapy. The FIRE-3 trial compared the efficacy of FOLFIRI plus cetuximab or bevacizumab in CRC based on the CMS classification and found that overall survival in CMS 3 and 4 subtypes were improved with FOLFIRI and cetuximab [66]. The CALGB/SWOG 80405 trial, which evaluated a similar chemotherapy regime and outcomes to the Fire-3 trial, found that overall survival in CMS 1 subtype patients benefitted from treatment with cetuximab while CMS 2 subtype patients benefitted from bevacizumab [67].

Importantly, 13% of CRC remains unclassified by the CMS system and is term “mixed”, suggestive of transcriptional ITH [64]. Árnadóttir et al. described their study in which multiregional biopsies from 14 CRC patients underwent RNA sequencing with CMS subtyping [68]. In about 20% of tumour biopsies, the authors found a large amount of transcriptional ITH between biopsies of the same tumour. GSEA of CRC with mixed transcriptional subtypes revealed genes responsible for cellular metabolism, cell cycle, Wnt signalling and immune response. Interestingly, the authors found that most mixed subtype tumours originated from the proximal colon.

While the complete biological impact of mixed-CMS subtype CRC needs to be evaluated, mixed subtype CRCs are likely to represent a phenotypically separate type of CRC as shown by clinical trials which have evaluated the response of CRC by CMS subtypes. In the LUME-Colon 1 phase III study of nintedanib in advanced CRC, mixed-subtype CRC demonstrated improved overall survival compared to CMS 2 or 4 [67].

## Interactions amongst subclones

There is uncertainty about the function and clinical significance of ITH in cancer [69]. A sudden explosion of genetic diversity akin to the “big bang” followed by a prolonged period of neutral evolution generates a heterogeneous distribution of mutations. These mutations form various subclone populations, which interact by competing and cooperating with each other. Although neutral evolution may give rise to the landscape of heterogeneity on an ecological level, the putative mechanism by which a premalignant phenotype switch to a malignant phenotype remains unresolved, and is likely to occur as a function of various subclone interactions within the tumour. In order to further explore the clonal interactions which occur at the earliest phases in the initiation of cancer, subclone interactions need to be studied.

### Clonal competition in cancer

Clonal competition recalls Darwinian evolutionary dynamics in which subclones gain an advantageous mutation which results in increased fitness relative to other subclones. Examples of clonal competition have been demonstrated in three recently published articles. Yum et al. utilised a mouse small intestine model with multicolour-labelled oncogene reporters. The authors found that *KRAS*^*G12D*^ and *PIK3CA*^*H1047R*^ mutant crypts modified the intestinal stem cell environment and primed stem cells towards differentiation. Compared to control mice, there was a larger proportion of differentiated cells relative to stem and transit-amplifying cells, leading to an increased fraction of mutant crypts in mutant mice. This clonal drift was likely mediated by paracrine BMP- and Wnt-mediated pathways [70]. In another article by van Neerven et al., the authors noticed a negative feedback loop in *APC* mutant organoids involving upregulation of WNT antagonist genes such as *Notum*, *Wif1*, and *Dkk2*. Secretion of these WNT antagonists into the paracrine environment appeared to result in competitive Wnt-pathway downregulation in wild-type cells, which was rescued when downstream activation of Wnt by GSK3β inhibitors were administered [71]. The competitive inhibition of wild-type cells by *APC* mutants was confirmed in another article by Flanagan et al. using a mouse model instead [72]. NOTUM inhibitor treatment resulted in reduced fixation of *APC* mutants.

Briefly, other examples include cells that gain the protein MYC causing the non-cell-autonomous activation of caspases which result in apoptosis of surrounding cells [73]. Another example includes mutations that target the Hippo pathway by negatively regulating the function of YAP/TAZ [74]. YAP/TAZ are transcriptional activators that enhance cell proliferation and may result in EMT. Mutations associated with disrupting the Hippo pathways give mutated cells a fitness advantage in cell growth [75]. These include increased Tead2 expression [76], as well as loss of the tumour suppressor gene *Scrib* [77].

This concept of clonal competition may seem at odds with the prevailing hypothesis that the evolutionary dynamics of cancer is one of neutral evolution. However, a model of clonal competition proposed by Colom et al. manages to resolve these two seemingly incompatible concepts [78]. Using an oesophageal cancer mouse model in which the normal oesophageal epithelium had been exposed to the mutagen diethylnitrosamine (DEN), mimicking tobacco smoking, for over a year, the authors observed that positively selected genetic mutants did not correlate with increased clone size. They evaluated a neighbour-constrained fitness model in which clonal competition existed between cells of varying fitness, but following a brief period, would exhibit neutral dynamics when cells of equivalent fitness encountered each other. *DN-maml1* cells represent a highly aggressive subclone. Using lineage-tracing experiments, they demonstrated that this subclone grew aggressively in the presence of normal epithelial cells but did not proliferate extensively when placed in epithelial cells which had gained varying mutations from prolonged DEN exposure. Moreover, they noted that although there was an initial growth advantage of *DN-maml1* cells in the DEN-exposed epithelium, this advantage decreased over time and reverted towards neutrality.

### Clonal cooperation in cancer

Subclones may instead interact in a cooperative way to enhance tumour growth. These include paracrine signalling as well as direct interactions between subclones. Hobor et al. were able to demonstrate the paracrine secretion of TGFα and amphiregulin by mutant *KRAS* cells on administration with cetuximab. When media from these cells were transferred to wild-type *KRAS* cells, a reduction in the effectiveness of cetuximab was observed [79]. In a similar study, chemo-resistant cells exposed to oxaliplatin secreted progranulin. CRC cells grown in conditioned media containing the progranulin secretions led to smaller tumour growths than those grown in media without secretions [80]. Paracrine methods of clonal cooperation have been observed in other cancers such as glioblastoma multiforme [81].

Another example of paracrine signalling in the CRC metastatic process includes cooperation between metastasising CRC cells and hepatocytes [82]. Normally, the hepatic microenvironment is hypoxic owing to perivenous hepatocytes which consume glycolytic substrates, such that CRC cells metastasising via the portal system undergo cell death. Subclones of CRC which exhibit miR-483-5p/miR-551a silencing and creatine kinase brain-type (CKB) overexpression are able to secrete CKB into the hepatic micro-environment, where hepatocyte derived creatinine can be converted to phosphocreatine. Phosphocreatine is then imported by CRC cells to generate ATP. Cooperation between CRC subclones and hepatocytes, therefore, allow the CRC cells to overcome hepatic hypoxic microenvironments and sustain metastasis.

Direct interactions between subclones have also been observed, albeit not in CRC. While studies providing evidence of direct interaction amongst CRC subclones remain forthcoming, the following examples illuminate possible mechanisms which might be observed in CRC. In these examples, subclones possessing one mutation couple with another subclone possessing a different mutation to either overcome individual subclone deficiencies or to result in a more proliferative phenotype,

In a mouse model of breast cancer, distinct subpopulations of *Hras*^*mut*^*Wnt1*^low^ and *Hras*^*wt*^*Wnt1*^high^ cells interacted with each other to produce a cancer phenotype [83]. The importance of clonal cooperation by *Wnt1*^high^ cells was evident when the *Hras*^*mut*^*Wnt1*^low^ subclone was transplanted into mice that possessed a doxycycline-inducible Wnt transgene. In the absence of doxycycline, tumour regression was observed, while tumour growth occurred only when doxycycline was re-administered. Interestingly, some tumours relapsed a few weeks later even in the absence of doxycycline. On examination of these tumours, it was observed that *Hras*^*mut*^ subclones were able to recruit heterologous Wnt-producing cells.

In a model of small cell lung cancer (SCLC) using primary mouse cancer, two subclonal populations from a common clonal origin were observed to interact in causing increased cellular proliferation [84]. One subclonal population expressed neuroendocrine markers such as synaptophysin (Syp), achaete-scute complex homologue 1(Ash1), and neural cell adhesion molecule (NCAM), while another subclone expressed mesenchymal markers and was low in NCAM. The authors observed that in vitro co-cultures of both subclones resorted to a much greater proliferation of cancer tissue. Moreover, phenotypic differences were seen between co-cultured and non-co-cultured cells, such that the phenotype which more accurately recapitulates a cancer phenotype was seen with the co-cultured cells. When cells were transferred in vivo into a mouse mode, abdominal metastases were marked in the co-cultured group.

## Non-genetic influences on tumour heterogeneity

While this review has focused on mutational evolution as a cause of phenotypic heterogeneity, this may also be controlled by mechanisms independent of intrinsic mutational background or clonal interactions. The transcription of genes governing cell identity, fitness and clonogenicity can be regulated by mechanisms such as epigenetics, hypoxia and interactions with the tumour microenvironment which are now discussed.

### Hypoxia

Hypoxia is brought about within the tumour due to rapid cellular proliferation resulting in increased diffusion distance from tumour cells to blood vessels, dysregulated angiogenesis, as well as a systemic reduction in oxygen-carrying capacity from anaemia [85]. Fluctuations of oxygen perfusion lead to cyclical hypoxia, which conversely promotes the survival of tumour cells via a hypoxia-inducible factor 1 (HIF-1) dependent manner during reoxygenation periods [86]. HIF-1 is a transcription factor that regulates genes controlling cell survival, angiogenesis and migration [87]. In hypoxia, HIF-1 binds to the *VEGF* promoter, leading to the expression of VEGF-A, an angiogenic protein that results in neovascularization [88]. In a convergence of driver pathways related to colorectal carcinogenesis, HIF-1 acts on many cancer-related genes. HIF-1 may be upregulated via the Ras-ERK pathway discussed previously in this review [89], and may also lead to activation of the Wnt/β-catenin pathway through the upregulation of WNT11 under hypoxic conditions [90, 91]. HIF-1 may also upregulate *TP53*, and in addition, form a HIF-1-p53 complex that mimics the activity of mutant p53 protein, even in the absence of the mutated gene [92].

### Epigenetic dysregulation

Dysfunction in DNA methylation can result in the suppression or upregulation of various genes, leading to tumour heterogeneity. DNA hypomethylation is associated with increased mitotic recombination, indel generation and chromosomal translocation, which may result in CIN [93]. In mice with only 10% expression of DNA methyltransferase 1 (Dnmt1), genome-wide hypomethylation resulted in a high incidence of chromosome 15 trisomy, leading to aggressive T cell lymphoma [94].

In contrast, hypermethylation may instead lead to tumour heterogeneity and cancer, as already described in an earlier section of serrated neoplasia in CRC. In CRC, CpG-island hypermethylation is associated with *MINT1*, *MINT2*, *MINT31*, *CDKN2A*, *hMLH1*, *CACNA1G*, *IGF2*, *NEUROG1*, *RUNX3*, and *SOCS1* [95, 96], and may be present in up to 20% of all CRCs [97].

### Epithelial cell—fibroblast interaction

The importance of cells in the stromal compartment in carcinogenesis arose from work classifying CRC according to gene expression patterns. Genes that were expressed in CMS 4 were discovered to be largely contributed by cells originating from the stromal compartment and not from epithelial cells [98]. These cancer-associated fibroblasts (CAFs) were shown to contribute to carcinogenesis by recruiting tumour-initiating cells via a TGF-β mediated pathway. Abnormal TGF-β signalling by stromal cells correlated with increased expression of stem-like and other poor prognostic gene sets in CRC patients.

CAF heterogeneity has been a topic of considerable interest in recent years, as the importance of the tumour microenvironment in carcinogenesis has been progressively described. The functional heterogeneity of CAFs is evident as some CAFs have been found to retard carcinogenesis while others promote it [99]. In a murine model of colitis-associated cancer, *Ikkβ* in Col1a2-expressing fibroblasts described a tumour suppressing the population of fibroblasts [100]. In another murine model, stromal Hedgehog pathway activity marked by the transcription factor GLI1 was responsible for the tumour retarding effect of this CAF subpopulation [101]. Kalluri described four subtypes of CAFs (F1 – F4) based on different roles which CAFs may play [102]. The F1 and F2 subtypes were described to be tumour-restraining and -promoting respectively. The F3 subtype were secretory fibroblasts, which have different roles based on the growth factor secreted by the fibroblast. The F4 subtype was able to remodel the extracellular matrix by affecting the tissue microenvironment. In a different approach, using ScRNA-seq, Li et al. revealed two subtypes of CAFs. The CAF-A subtype expressed genes that were related to extracellular matrix remodelling, reminiscent of the F4 subtype, while the CAF-B subtype expressed genes associated with the cytoskeleton and activated myofibroblasts [103].

Further studies have demonstrated the extensive crosstalk which occurs between epithelial cells and the stromal compartment. A study by Ouahoud et al. elucidated a feedback loop between epithelial and stromal cells [104]. In this study, it was found that SMAD4-deficient colorectal epithelial cells secreted a cytokine of the TNF ligand family to induce bone morphogenic protein 2 (BMP2) production by CAFs. BMP2 secreted by CAFs, in turn, lead to increased invasion and metastasis, resulting in poorer overall survival in patients.

### Epithelial cell—immune cell interaction

Apart from epithelial cells and fibroblasts, tumour-infiltrating immune cells (TIICs) make up a significant component of cancer tissue. Types and densities of TIICs have been associated with prognosis and response to treatment in CRC [105]. At present, there is a widely accepted consensus that tumour progression is influenced by a constant interplay between epithelial cells and the immune system [106].

Interactions between epithelial cells and a diverse array of immune-related cells have been described in the literature. The role of checkpoint inhibitor proteins such as PD-L1 in inhibiting T-cell function has already been discussed earlier in this review. Cooperation between epithelial cells and macrophage populations may also give rise to increased proliferation and enhanced tumour cell survival. Macrophages are stimulated to produce IL-1β [107]. IL-1β enhances Wnt signalling in tumour cells by inactivating GSK3β, a key component of the β-catenin destruction complex. Immunohistochemical analysis of tumour infiltration by CD8^+^ and CD57^+^ natural killer (NK) cells were also found to be associated with improved prognosis in CRC [108].

Unsurprisingly, dendritic cells have been found to play a critical role in CRC. As antigen-presenting cells, dendritic cells play an important role in bridging between the innate and acquired immune systems by presenting antigen to T-cells. One mechanism by which tumour cells may seek to decrease the activity of dendritic cells is by blocking the maturation of dendritic cells [109]. CD83^+^ mature dendritic cells were found to be decreased in the tumour microenvironment of patients in advanced stages of CRC [110]. Intriguingly, maturation of dendritic cells could be induced when cultured in media derived from human CRC tissue via an IL-12p70 inhibitory pathway [111].

Characterising interactions between CRC epithelial cells and the immune compartment is still in its infancy and our understanding of detailed mechanisms of interactions remains at an early stage.

## Conclusion

Vogelstein’s adenoma-carcinoma sequence highlighted the central role which driver mutations play in CRC. These driver mutations control important pathways, such as nuclear translocation and activation of transcription factors, or controlling the cell cycle, and can lead to cell growth and proliferation. The discovery that tumours exhibited high levels of ITH, however, suggests that a linear accumulation of driver mutations incompletely describes the carcinogenic process. Moreover, the composition of ITH within premalignant lesions and malignant differs. In fact, many driver mutations may already be present in premalignant lesions, yet, do not manifest as cancer. Computational modelling has assisted in our understanding of ITH and the evolutionary mechanics behind carcinogenesis and has suggested that an explosion of genetic diversity is created early in a premalignant lesion, followed by a period of neutral evolution, possibly punctuated with catastrophic gene-altering events.

Regardless, questions remain unanswered. Given that premalignant lesions already harbour driver mutations, the precise mechanism by which these malignant genotypes manifest as a malignant phenotype is unknown. There is evidence of clonal competition and cooperation in mouse models of cancer, with evidence that these interactions result in a malignant phenotype. Further research will need to be performed using human tissue models to further characterise the in vivo mechanisms that drive carcinogenesis.
